# Polyamide-MIL-101(Cr) Thin Films Synthesized on Either the Outer or Inner Surfaces of a Polysulfone Hollow Fiber for Water Nanofiltration

**DOI:** 10.1021/acsami.0c21571

**Published:** 2021-02-03

**Authors:** Carlos Echaide-Górriz, Yolanda Aysa-Martínez, Marta Navarro, Carlos Téllez, Joaquín Coronas

**Affiliations:** † Instituto de Nanociencia y Materiales de Aragón (INMA), Universidad de Zaragoza-CSIC, 50018 Zaragoza, Spain; ‡ Chemical and Environmental Engineering Department, Universidad de Zaragoza, 50018 Zaragoza, Spain; § Advanced Microscopy Laboratory (LMA), Universidad de Zaragoza, 50018 Zaragoza, Spain

**Keywords:** interfacial polymerization, thin film nanocomposite, hollow fiber, nanofiltration, metal−organic framework, microfluidics

## Abstract

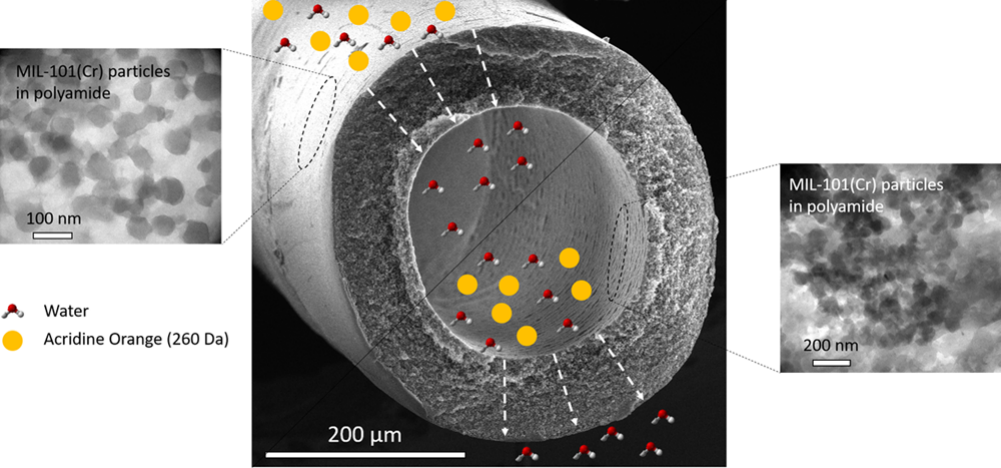

High-performance thin film nanocomposite (TFN) hollow fiber (HF) membranes, with MIL-101(Cr) MOF nanoparticles (52 ± 13 nm) embedded, have been synthesized with the polyamide layer formed either on the outer or inner surface of a polysulfone HF (250 and 380 μm ID and OD, respectively). The TFN_out membrane was developed using the conventional interfacial polymerization method, typically applied to obtain TFN flat membranes (MOF particles added to the thin layer by deposition). This membrane gave a water permeance value of 1.0 ± 0.7 L·m^–2^·h^–1^·bar^–1^ and a rejection of 90.9 ± 1.2% of acridine orange (AO, 265 Da). In contrast, the TFN_in membrane was synthesized by microfluidic means and gave a significantly higher water permeance of 2.8 ± 0.2 L·m^–2^·h^–1^·bar^–1^ and a slightly lower rejection of 87.4 ± 2.5% of the same solute. This remarkable increase of flux obtained with small solute AO suggests that the HF membranes developed in this work would exhibit good performance with other typical solutes with higher molecular weight than AO. The differences between the performances of both TFN_in and TFN_out membranes lay on the distinct superficial physicochemical properties of the support, the synthesis method, and the different concentrations of MOF present in the polyamide films of both membranes. The TFN_in is more desirable due to its potential advantages, and more effortless scalability due to the microfluidic continuous synthesis. In addition, the TFN_in membrane needs much fewer quantities of reactants to be synthesized than the TFN_out or the flat membrane version.

## Introduction

Nanofiltration is a process that aims at separating different mixtures that involve water and organic solvents, as well as ionic solutes and organic molecules with molecular weights between 200 to 1000 g·mol^–1^, by economic and efficient means. Many researchers from several countries have studied and suggested different membrane structures, among which the thin film composite (TFC) and nanocomposite (TFN) membranes are two of the most successful types.^1^ The structure of these membranes, which consists of an asymmetric support with a selective thin layer of polyamide (PA) on top, allowed to change the physicochemical properties of each layer separately.^2^ For this reason, many combinations of polymers have been studied,^3^ several of them available as commercial membranes.

Cadotte et al.^4^ pioneered the synthesis by interfacial polymerization (IP) of the first TFC membrane in 1980, while Jeong et al. prepared the first TFN in 2007.^5^ The latter achieved the combination of a TFC membrane with embedded zeolite nanoparticles (NPs) intending to improve the permeance in reverse osmosis without lowering the salt rejection. In 2013, Sorribas et al.^6^ developed metal–organic framework (MOF)-embedded TFN membranes for organic solvent nanofiltration (OSN) with enhanced separation properties because of the high specific surface areas, narrow porosity, and inorganic–organic character of these nanostructures (MIL-101(Cr), ZIF-8, MIL-53(Al), and NH_2_-MIL-53(Al)) for good compatibility with polymers. Later on, several authors studied the effect of other MOF NPs (MIL-68(Al) and ZIF-11,^7^ and UiO-66, ZIF-8, and ZIF-93,^8^ and the simultaneous combination of two complementary MOFs (ZIF-11 and MIL-101(Cr)^9^) in the performance of TFN membranes for OSN.

However, all these researchers used flat sheet membranes. A few authors studied the IP method to yield TFC-hollow fiber (HF) membranes and TFC-tubular membranes because of their higher intensification and productivity, given by the higher membrane area per cubic meter of membrane module they offer as compared to flat membranes.^10^ Here, two possibilities came up: the creation of the PA thin film on the outer (TFC_out) surface of the HF or its synthesis on the lumen side (TFC_in). Parthasarathy et al.^11^ synthesized a TFC_out, and Liu et al.,^12^ An et al.^13^ and Rajaeian et al.^14^ further developed optimized versions of this structure using particles of SAPO-34, ETS-4, and TiO_2_ to synthesize TFN membranes. In contrast, the first TFC_in was synthesized by Veríssimo et al.^15^ Continuing this line, some other authors added different types of nanoparticles into the PA thin film (giving rise to TFN_in membranes). Gai et al.,^16^ for instance, developed a TFN_in membrane combined with Na^+^ carbon quantum dots (NaCQD) using a polyethersulfone (PES) support. Lin et al.^17^ synthesized TFN_in membranes including dopamine functionalized HKUST-1 for brackish water filtration supported on a PES HF (0.9 mm ID). Urper-Bayram et al.^18^ fabricated a TFN_in membrane combined with TiO_2_ NPs using a multiwalled carbon nanotube modified polysulfone as support to filtrate MgSO_4_ and NaCl from water. Plisko et al.^19^ added fullerenol (C_60_(OH)_22–24_) to an inner PA thin film as an antifouling method. Additionally, Ingole et al.^20^ and Baig et al.^21^ recently prepared TFN membranes for gas dehydration based on MOF NH_2_-MIL-125(Ti) and acid-activated bentonite and TiO_2_ nanoparticles, respectively. Table 1 summarizes the main advantages and drawbacks of the two approaches presented in this paper (TFC_in/TFN_in and TFC_out/TFN_out HF membranes).

**Table 1:** Main Advantages and Drawbacks of IP on the Outer or Inner Surface of an HF Asymmetric Support

surface	advantages	drawbacks
outer	similar to flat supports with total access for characterization (e.g., of crystalline NPs in case of TFN membranes)	skin layer unprotected
	ease of possible industrial up-scale	creation of dead volumes between HFs in a membrane module in operation
inner	use of microfluidics to save reactants and NPs (in the case of TFN membranes) and allow controlled reaction and eventual sequencing for post-treatment	difficult access at small inner diameters giving rise to clogging and limiting the use of agglomerating NPs
	protected skin layer	up-scale can be challenging
	high control of flow and mass transfer for precise synthesis of the selective layer	difficult access for characterization
	accessible to up-scale since the feed of every HF is wholly isolated from each other	high drop-pressure

In line with these studies, in this work, TFN membranes have been successfully synthesized embedding MIL-101(Cr) NPs either on the outer or on the inner surface of polysulfone HFs. This well-known MOF was chosen because it showed interesting effects in flat TFN membranes for OSN^6,9,22^ due to its hydrophilic character, wide specific surface area with pore apertures of 1.2 and 1.6 nm, and high crystallinity.^23^ The TFN_in and TFN_out HF membranes were compared to their homologous TFC_in and TFC_out membranes to study the consequences of the creation of the thin film on the outer or inner surface of the HF and the impact of the MOF on the structure and separation performance. All membranes synthesized were characterized not only by SEM and TEM but also by chemical detection techniques such as EDX, STEM-EDS, and XPS, so that the presence of crystalline MOF NPs in the samples was fully proved.

## Materials and Methods

### MOF Particles Synthesis

MIL-101(Cr) NPs were crystallized following a hydrothermal synthesis procedure:^24^ 0.70 g of CrCl_3_·6H_2_O (≤ 98%, Sigma Aldrich) and 0.45 g of terephthalic acid (98%, Sigma Aldrich) in 26 mL of deionized water. The obtained solution was heated at 180 °C for 30 min in a microwave (Anton Paar, Multiwave 3000). The synthesized nanocrystals were activated as follows: first, they were washed and centrifuged at 10,000 rpm for 15 min with deionized water. Second, the MOF NPs were treated with DMF (99.5%, Scharlau) at 200 °C for 24 h. Finally, they were washed overnight with methanol (99.9%, Scharlau) at 70 °C with two stages of washing and centrifugation at 10,000 rpm for 15 min with methanol.

### HF Supports

The membrane manufacturer Polymem Fabricant de Membranes kindly supplied the polysulfone (PSf) HF supports. This company designed this type of membrane for microfiltration processes with external pore sizes of about 200 nm. The inner and outer diameters (ID and OD) are 250 and 380 μm, respectively. A membrane module with a volume of 1 m^3^ built with the fibers used in our study would achieve m^2^·m^-3^ ratios of ∼6900 and ∼10,500 when referring to the internal and external HF surfaces, respectively.

### TFC and TFN HF Membrane Synthesis

The interfacial polymerization (IP) method consists of the reaction between two monomers, *m*-phenylenediamine (MPD, 99%, Sigma Aldrich) and trimesoyl chloride (TMC, 98%, Sigma Aldrich) in the interface between two immiscible solvents, giving rise to an aromatic polyamide thin film.

**Figure 1:**
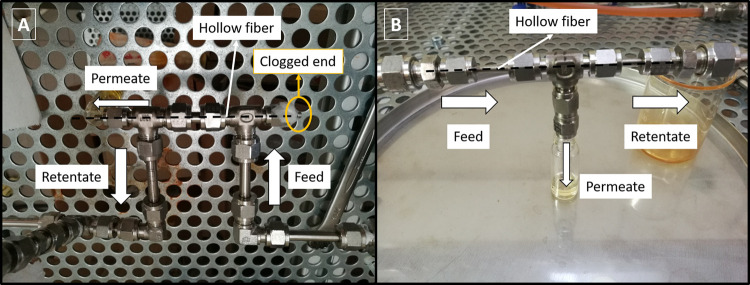
Membrane modules for permeation from the shell side to the lumen side (A) and from the lumen side to the shell side (B).

For the synthesis of the thin film on the outer surface of the HF, a method recently published was followed.^25^ We first immersed a piece of HF 12 cm long in an aqueous solution with a 2% (w/v) of MPD for 2 min. After that, the excess solution was removed with tissue paper. Then, the HF was immersed in an organic solution composed of 0.1% (w/v) of TMC in *n*-hexane (extra pure, Scharlab) for 1 min, forming the polyamide. Finally, the rest of the organic solution was removed using fresh *n*-hexane and that of MPD using deionized water. To obtain a TFN membrane, the MIL-101(Cr) NPs must be dispersed in the organic solution before the IP occurred. The organic solution would then be composed of 0.1% (w/v) of TMC and 0.2% (w/v) of MIL-101(Cr) NPs (see Table 2). The amount of MIL-101(Cr) NPs was the same as that applied as optimum in the flat TFN membranes developed by Sorribas et al. a few years ago.^6^ The TFC or TFN membrane would then be placed in a stainless steel membrane module (see Figure 1A), sealing both ends with an epoxy resin (Araldite).

**Table 2:** Synthesis Parameters^a^

		aqueous phase			organic phase (*n*-hexane)		
membrane	description	*C* % (w/v)	t* (min)	*Q* (μL·min^–1^)	*C* % (w/v)	t* (min)	*Q* (μL·min^–1^)
TFC_out	PA film on the outer surface	2	2		0.1 TMC	1	
TFC_in	PA film on the lumen	2	5	70	0.3 TMC	1.5	70
TFN_out	PA film + MIL-101(Cr) on the outer surface	2	2		0.1 TMC + 0.2 MIL-101(Cr)	1	
TFN_in	PA film + MIL-101(Cr) on the lumen	2	5	70	0.3 TMC + 0.2 MIL-101(Cr)	1.5	70

a *C*, concentration (MPD in the aqueous phase, TMC in the organic phase, with 0.2% (w/v) of MOF NPs in the case of TFN membrane synthesis); *t*, time; *Q*, feed flow. *, contact time between the aqueous or organic phase and the support.

IP assisted by microfluidic means was applied to synthesize the PA thin film on the inner surface of a 12 cm long HF support, as we did in a previous research related to TFC and MOF membranes.^25,26^ Using a syringe pump, the MPD solution, whose composition was identical to that used in the previous synthesis (2% w/v), was fed to the fiber inside at a rate of 70 μL/min for 5 min. Pure cyclohexane (Scharlab, extra pure) was then pumped with a different syringe pump at a rate of 157 μL/min for 1 min to remove the excess MPD solution from the lumen side. After that, a solution of 0.3% (w/v) of TMC in *n*-hexane was pumped with a third syringe pump at a rate of 70 μL·min^-1^ to start the PA formation. The lumen side was finally washed successively with *n*-hexane and deionized water. To obtain the TFN membrane, the MIL-101(Cr) NPs must be dispersed in the organic solution at a concentration of 0.2% (w/v). Once the TFC or TFN membranes were obtained, they were mounted on the membrane module shown in Figure 1B, sealing both ends with an epoxy resin (Araldite).

Three samples per membrane type were synthesized, and thus averages and standard deviations of both water permeance and dye rejection values could be calculated.

### Characterization

The crystallinity of the MIL-101(Cr) NPs was confirmed by X-ray diffraction (XRD) measurements. The results obtained in the experiments were comparable to simulations obtained from ref (23). The measurements were carried out in a D-Max 2500 Rigaku diffractometer with a Cu Kα (λ = 0.15418 nm) rotating mode, from 4 to 40° (2θ) with a 0.025° s^–1^ step, operated at 40 kV and 80 mA.

Scanning electron microscopy (SEM) was applied to observe the fabricated HF membranes. Different areas in each type of membrane were observed to obtain a qualitative estimation of the MOF NP content. The cross-section area of the TFN_out was analyzed for atomic composition, and thus it was possible to observe the MOF NPs into the porosity of the membrane. For that purpose, the membrane was freeze-fractured in liquid N_2_. Energy-dispersive X-ray (EDX) microscopy was useful to quantify the elements that form the thin film in the areas previously seen in SEM. Samples were coated with 14 nm of Pd. The equipment used was an FEI-Inspect F50 microscope at an acceleration voltage between 10 and 20 kV with a spot size of 2.5 and 3.5 nm.

Thermogravimetric analysis (TGA) was used to calculate the thermal stability of MIL-101(Cr) and to determine whether its porosity was adequately activated. The measurements were taken in a Mettler Toledo TGA/SDTA 851e system, using an air atmosphere and a heating rate of 10 °C·min^–1^, until 700 °C.

Transmission electron microscopy (TEM) of bare MIL-101(Cr) NPs and MIL-101(Cr) NPs embedded in PA thin film was performed using an FEI Tecnai T20 microscope, operated at 200 kV. Using this technique, the NP average size was estimated, as well as checked the distribution and morphology of MIL-101(Cr) NPs within the PA from TFC_in and TFC_out membranes. A sample of either a TFN_in or TFN_out membrane was immersed in DMF for approximately 10 min dissolving the polysulfone support, and then the MOF-PA (non-soluble in DMF^27,28^) thin film detached from it. The film was placed onto a carbon-coated 300 mesh copper grid and allowed to dry for 48 h under ambient conditions. Finally, in the areas observed by TEM, electron diffraction (ED) was performed to prove the MOF crystallinity after the IP process. Furthermore, a TFN_in membrane was embedded in an epoxy resin (EMBed 812) at 60 °C for 24 h and sectioned using an ultramicrotome Leica EM UC7. Ultrathin sections of about 70 nm thick were obtained and analyzed at 200 kV to measure the PA film thickness, structure, and arrangement over the PSf HF support.

Scanning transmission electron microscopy (STEM) and X-ray spectrometry (EDS), corresponding to an FEI Tecnai F30 microscope at 300 kV, were required to detect the main elements that confirm the presence of MIL-101(Cr) in the thin film detached from the TFN_out and TFN_in membranes (the same sample used for the previous TEM imaging). The EDS was useful to quantify the elements detected, and also the STEM imaging itself can highlight the areas where metals are present because of the contrast differences dependent on the atomic numbers of the different components (heavier elements would appear highlighted in a lighter grey, in contrast to the more blackish lighter elements).

X-ray photoelectron spectroscopy (XPS) experiments were conducted to quantify the amount of carbon (C), oxygen (O), and nitrogen (N) in the PA thin films of TFN_out membranes. In the case of the TFN_in membrane, the sample was the grid prepared for the TEM and STEM characterization indicated above in order to avoid the signal from the polysulfone support, easier to elude in the TFN_out membrane configuration. The XPS characterization was performed with a Kratos Axis Ultra spectrometer, using a monochromatic Al Kα (1486.6 eV) X-ray source at 10 mA and 15 kV and a power of 150 W. The samples were first air evacuated at room temperature (and at pressures near 10^–11^ bar) and analyzed in 0.7 × 0.3 mm^2^ areas under the same conditions. With the information gathered, the C/N and O/N ratios, which can be related to the cross-linking degree of the PA, were calculated for both TFN membranes. The amount of chromium (Cr) was also measured and applied to estimate the MOF content using the empirical formula of MIL-101(Cr) as previously done in other studies.^29^

Atomic force microscopy (AFM) was applied to measure the roughness on both the outer and inner surfaces of the HF supports. The equipment used was a VEECO Multimode 8 with a tapping mode used in ambient air conditions together with a single crystal silicon antimony-doped cantilever provided by NT-MDT Spectrum Instruments. The method to carry out these measurements on the outer surface was straightforward since the cantilever can have easy access to this surface. The AFM measurements on the inner surface needed a more sophisticated method. A bunch of five 2 cm-long bare support samples was put together and then embedded in epoxy resin (Araldite) to obtain a piece of 3 × 1 × 1 cm. Then, slices were cut using a cutter. With this procedure, it was possible to access easily to the lumen of the HFs. Three different areas of 10 × 10 μm in size were observed on the outer surface of the substrate, and the roughness value was measured and averaged. On the inner surface, in contrast, areas of different dimensions were observed: 30 × 30 μm, 10 × 10 μm, and 4 × 4 μm. From these measurements, both average roughness and 3D models of the surface were obtained.

### Nanofiltration Experiments

The modules prepared contained only one fiber each. As the inner and outer diameters of the HFs were 250 and 380 μm, respectively, the modules prepared (8 cm long) with the TFC and TFN membranes had respective active surfaces of 3.0 × 10^–5^·m^2^ in case of filtration from the lumen to the shell side (Figure 1B) and 4.7 × 10^–5^·m^2^ in case of filtration from the shell to the lumen (Figure 1A).

A cross-flow filtration installation, whose scheme can be seen in Figure S1, was used for the nanofiltration tests. The feed was an aqueous solution with acridine orange (AO, 265 Da) as solute (20 mg·L^-1^) in a continuous flow configuration at 6 bar and 20 °C. The experiments lasted for 6 h, measuring both permeance and rejection (see eqs 1 and 2, respectively) every hour.
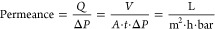
1
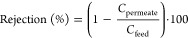
2where *Q* is the permeate flux, Δ*P* is the pressure gradient, *V* is the volume of permeate collected in a given time *t*, *A* is the membrane area, different at every membrane side, and *C*_permeate_ and *C*_feed_ are the solute concentration in both permeate and feed. A Jasco V-670 UV–vis spectrophotometer was used, previous calibration, to obtain the AO concentration at 480 nm as the wavelength of maximum absorbance.

## Results and Discussion

### MIL-101(Cr) Characterization

**Figure 2:**
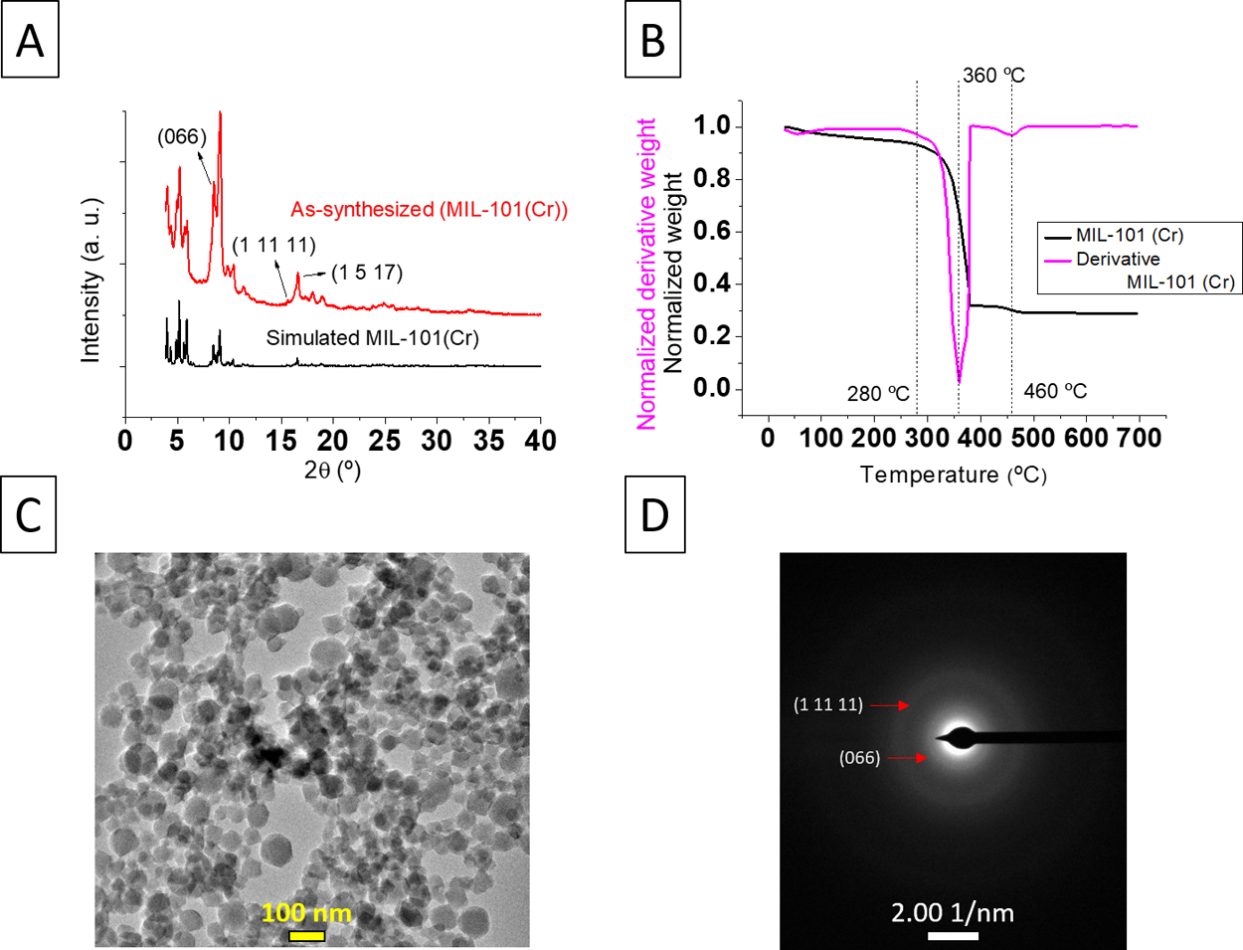
Experimental and simulated XRD patterns of MIL-101(Cr) (simulated XRD taken from ref (23)) (A); TGA curves of MIL-101(Cr) (B); TEM image of MIL-101(Cr) (C); ED of the same area, with two of the MIL-101(Cr) characteristic crystallographic planes evidenced (066 and 1 11 11) (D).

MIL-101(Cr) crystalline NPs were achieved according to their XRD pattern (see Figure 2A).^23,30,31^ Additionally, the TGA curve shows the total activation of the MIL-101(Cr) in agreement with the lack of mass losses prior to the degradation temperature (see Figure 2B), except for a 7% lost at the beginning of the curve, probably due to the well-known hydrophilicity of this MOF.^6^

Additionally, the morphology of the NPs observed in the TEM image (see Figure 2C) seems to be similar to those of previous publications,^6,29^ and the ED confirmed that they were MIL-101(Cr), as it evidenced the presence of the (066) and (1 11 11) diffraction planes of the MOF (see Figure 2D). These diffractions correspond to d-spacings of 10.5 and 5.7 Å, respectively. The particle size of the MIL-101(Cr) is 52 ± 13 nm, adequate to form a continuous and selective PA film with well-dispersed, embedded MOF NPs.

### Membranes Characterization. SEM and EDX Mapping

#### HF Support

**Figure 3:**
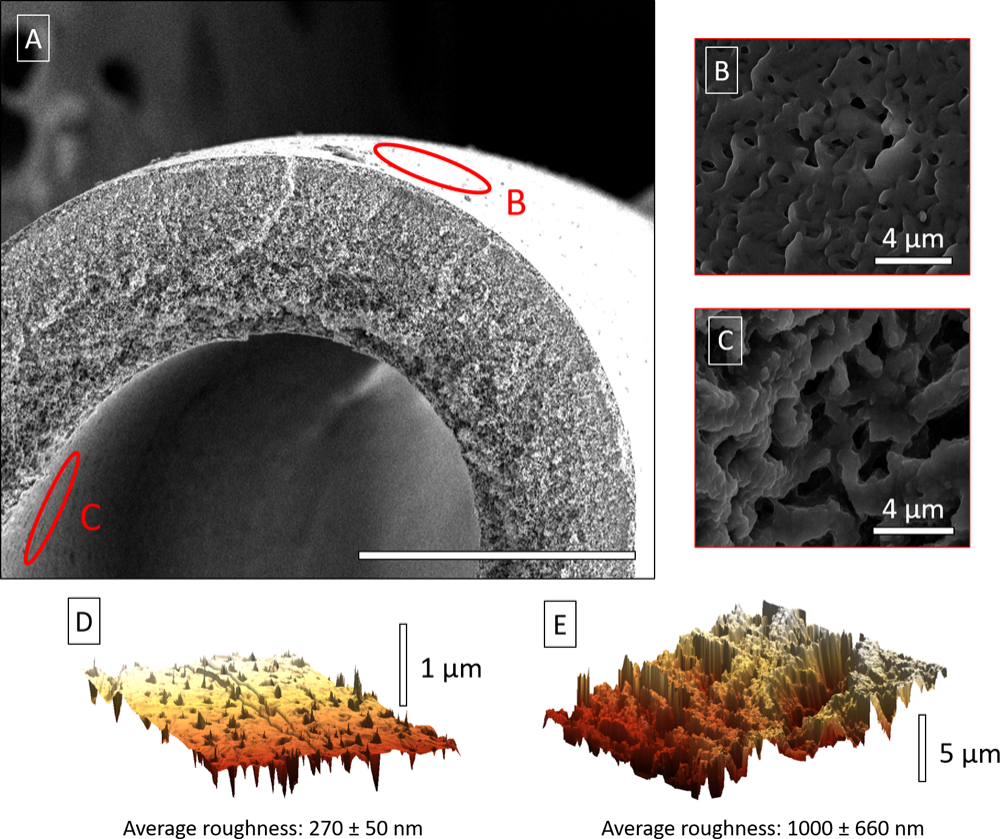
SEM images of HF (A) and its outer (B) and inner (C) surfaces, both of them highlighted in (A). Roughness 3D model of a 10 × 10 μm area of the outer (D) surface and a 30 × 30 μm of the inner surface (E). See the three areas explored on each side by AFM in Figure S2.

The PSf HF used as support presents morphological differences in superficial pore size and roughness between its outer and inner surfaces. While the pores on the outer surface have diameters of 950 ± 260 nm, those on the inner surface have diameters of 2700 ± 1200 nm (see Figure 3A–C). Similarly, the inner surface is rougher than the outer surface (an average roughness of 1000 ± 660 nm compared to 270 ± 50 nm), as it can be seen in the AFM 3D models of Figure 3D,E. Even if the current work is focused on only one type of support, especially suitable due to its commercial application and availability, the influence of the support on the synthesis of TFC membranes has been addressed by several authors from the point of view of porosity and hydrophobicity.^25,32^ One of the key issues deals with its chemical composition, while for water nanofiltration applications, PSf are suitable, and in the case of organic solvent nanofiltration, solvent-resistant polymers submitted to cross-linking are applied.^2,3^

#### TFN_out Membrane

**Figure 4:**
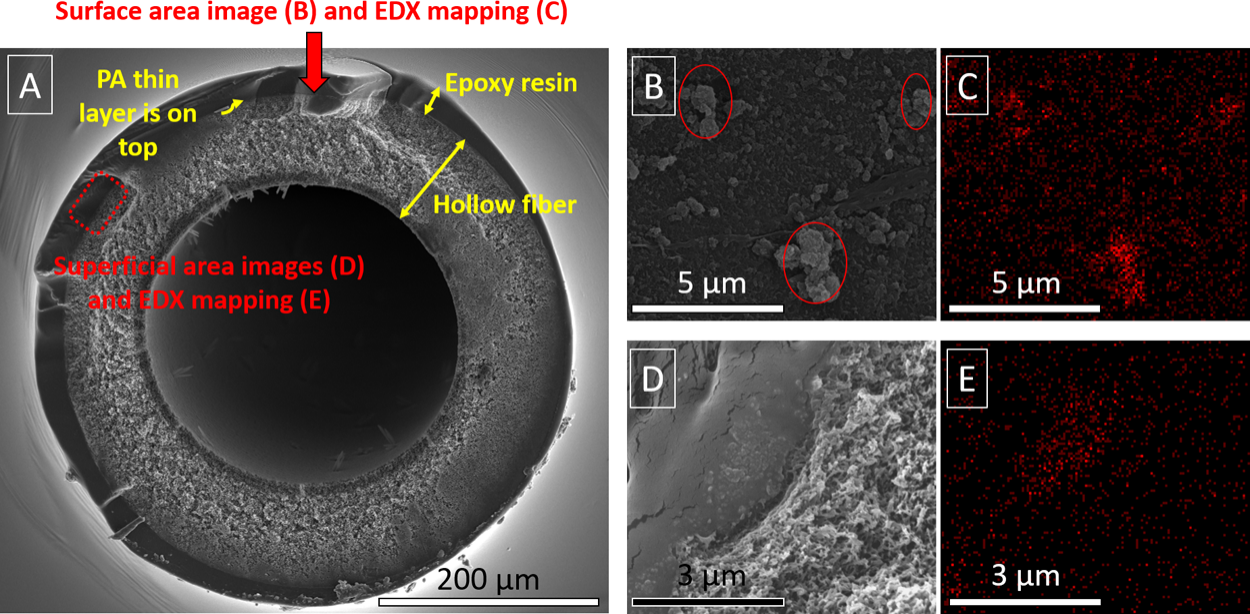
SEM and EDX characterization of HF membrane TFN_out: cross-section area; this image is a scheme that shows where the SEM images and EDX mappings were obtained (A). SEM image of the surface of the TFN_out membrane with the MOF agglomerates highlighted in red (B). EDX mapping of the surface in (A) with the Cr atoms in red (C). SEM image of the cross-section area (D). EDX mapping of the cross-section area in (D) with the Cr atoms in red (E).

Figure 4A shows an overview of the areas observed in the TFN_out membrane where the HF and the epoxy resin (used to prepare the sample) thicknesses are highlighted. Additionally, the areas where the superficial images and the cross-section images were taken are marked in red. The SEM image of the TFN_out membrane depicts three agglomerates of MIL-101(Cr) (highlighted in red circles, see Figure 4B), surrounded by the typical ring-like shapes of the PA thin film.^29^ The EDX mapping evidenced the presence of Cr atoms, mainly concentrated in the highlighted areas of Figure 4B (see Figure 4C), and some disperse red dots in spaces between them, where no NPs are observable with the naked eye. Since the electron beam penetrates several micrometers into the sample during EDX characterization, not all atoms detected are necessarily present at the membrane surface but at different depths. The SEM images of the cross section corroborate this hypothesis (see Figure 4D): a 1 μm-thick mass, more significant than the 50–100 nm selective thin film. The corresponding EDX mapping (see Figure 4E), where the red dots represent the Cr atoms, confirmed that some of those MOF NPs penetrated into the support.

In conclusion, the presence of MIL-101(Cr) NPs was evidenced in the thin film. Even though some MOF agglomerates were found in the previous images, there are dispersed dots in the EDX mappings evenly distributed along the membrane surface and thickness. Therefore, the MIL-101(Cr) NPs are likely quite well dispersed along the three dimensions of the PA thin film.

#### TFN_in Membrane

**Figure 5:**
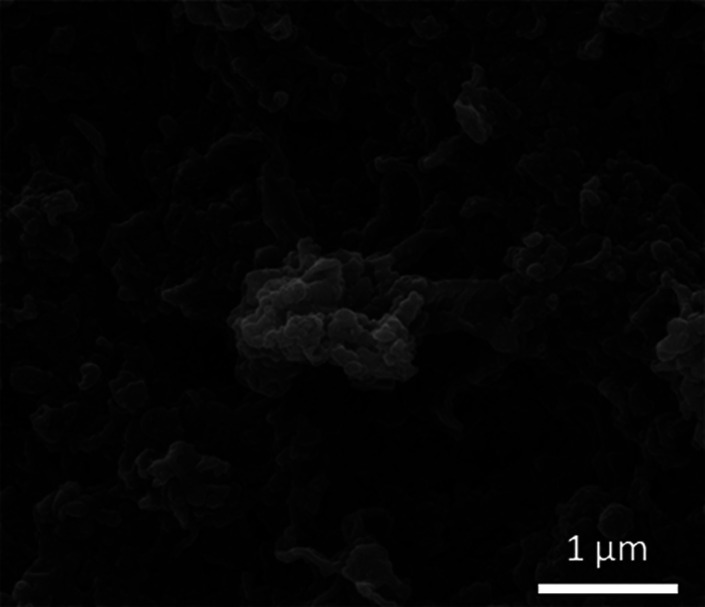
SEM image of the surface on the lumen of the TFN_in membrane.

In this case, MIL-101(Cr) NPs or their corresponding aggregates were not sufficiently concentrated to be detected by their chromium content using EDX analysis or SEM images. The SEM micrograph in Figure 5 only shows the same ring-like structures of the PA found in Figure 4B.

#### TEM, ED, and XPS of Membranes and Detached Films

**Figure 6:**
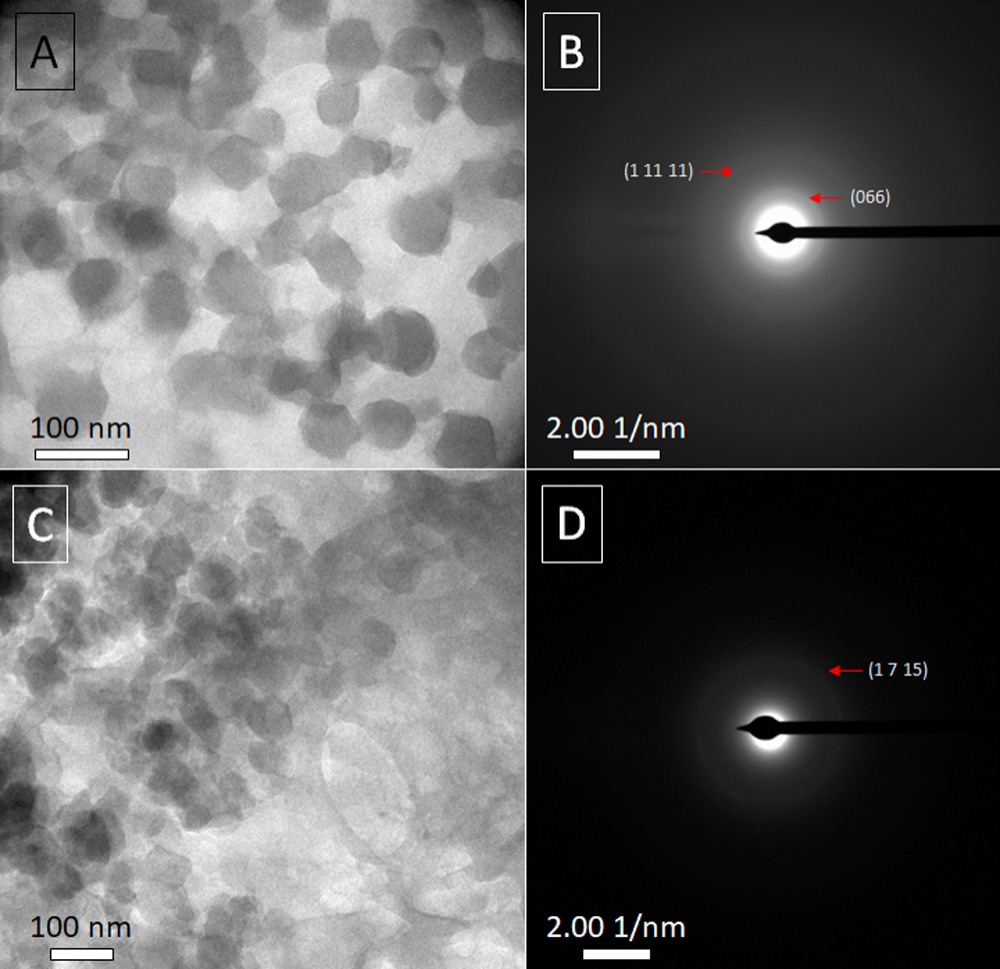
TEM image of the detached thin film obtained through the dissolution of the PSf support of TFN_out membrane (A). ED spots corresponding to panel A (B). TEM image of the detached PA thin film with MIL-101(Cr) NPs of TFN_in membrane (C). ED spots corresponding to panel C (D). Red arrows point to spots consistent with MIL-101(Cr) characteristic crystallographic planes.

To prove the presence of crystalline MIL-101(Cr) NPs directly on the PA thin film, the PA with embedded MOF NPs from both TFN_in and TFN_out membranes was analyzed by TEM following the procedure described in the experimental section (removal of the PSf support using DMF as solvent, thereby the isolated PA thin film can be deposited on a TEM copper grid). TEM imaging allowed to observe MIL-101(Cr) NPs wrapped in a grey amorphous mass of PA in both types of PA thin membranes (see Figure 6A,C). This characterization shows that MIL-101(Cr) NPs retained their typical morphology^24^ after the IP process, as previously reported.^6,29^

Moreover, the ED image of the thin film detached from the TFN_out membrane (Figure 6B) shows two diffraction rings that can be indexed as the (066) and (1 11 11) planes (d-spacings of 10.5 and 5.7 Å, respectively) of MIL-101(Cr) NPs. The intensity of those diffraction rings is weak because of the crystal degradation that most MOFs suffer due to their electron beam-sensitive nature and the amorphous PA thin film that covers the MOF NPs. Therefore, it can be assumed that the crystal structure of MOF NPs was maintained after the IP process.

The same results were obtained from the analysis of the PA thin film detached from the TFN_in membrane: the TEM imaging (Figure 6C) allowed to evidence some MIL-101(Cr) nanoparticles dispersed in the PA (grey mass around), and the ED confirmed it (see the ring corresponding to the (1 7 15) diffraction plane in Figure 6D, with a d-spacing of 5.2 Å). Those diffraction rings were observed upon analyzing bare MIL-101(Cr) nanoparticles (see Figure 2D) and they served us to confirm the fact that the MIL-101(Cr) NPs were embedded into the thin films of both membrane types and retained their crystallinity.

**Figure 7:**
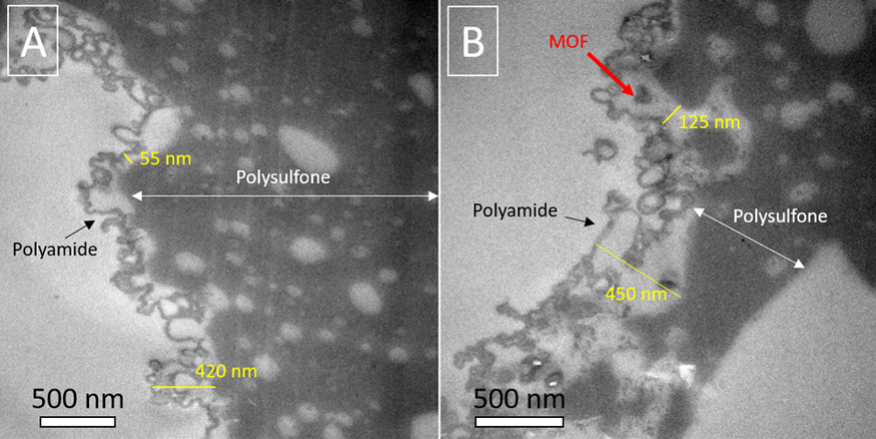
TEM images of cross-sections of TFC (A) and TFN_in (B) with MOF NPs embedded, where the red arrow indicates the possible location of an MIL-101 (Cr) NP (∼70 nm in size).

Figure 7 shows cross-section TEM images of the TFC membrane and TFN_in membrane with MOF NPs embedded in the PA film (Figure 7A and Figure 7B, respectively). This specific TEM characterization was carried out only on the most relevant TFC_in and TFN_in membranes but not on the TFC_out and TFN_out membranes of the worst nanofiltration performance (see below). TFC and TFN_in thicknesses are heterogeneous in the approximately 55–420 and 125–450 nm ranges, respectively. These thicknesses depend on the PA structure and whether MIL-101(Cr) NPs have been sectioned embedded in the PA film or not. The PA film in this membrane present a pronounced ridge-and-valley structure that have been formed on the inner-surface of the polysulfone HF support, where bigger and more interconnected pores (Figure 3C) and a rougher surface (Figure 3E) are present. This, together with the higher water permeances achieved with the TFN_in membrane as compared to those of the TFN_out one (see below), suggests that the PA film was thicker in the TFN_in membrane than in the TFN_out membrane.

The final characterization technique used to analyze the detached thin films was the STEM imaging combined with EDS. Figure S3A,B evidenced the dispersion of Cr atoms on both samples. Nevertheless, those atoms, known to belong to MIL-101(Cr) NPs, are distributed in located areas, probably occupied by MOF NPs. The EDS spectra that evidenced the presence of Cr on the areas of Figure S3 are available in Figure S4.

#### Nanofiltration Tests

**Figure 8:**
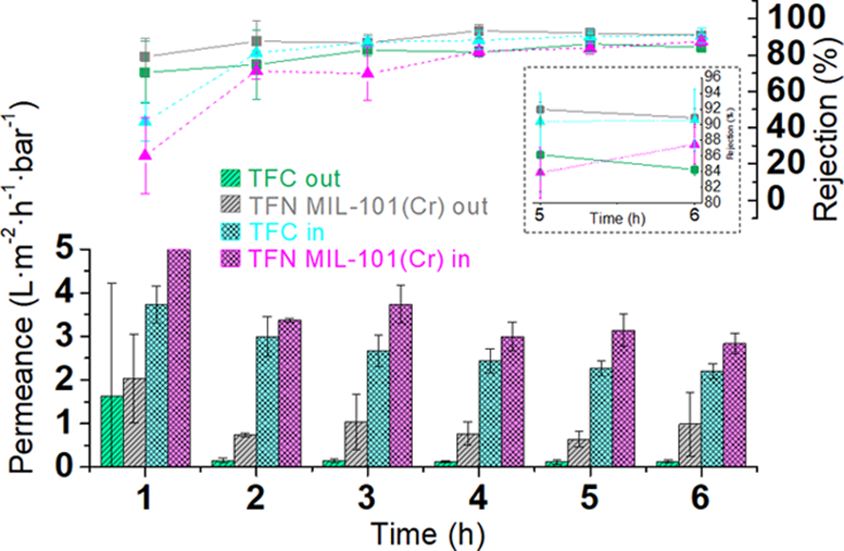
Permeances and rejections of the TFC_out membrane (green), TFN_out membrane (grey), TFC_in membrane (light blue), and TFN_in membrane (purple). A closer view of rejections at 5 and 6 h is included in the inset. The nanofiltration tests were carried out at 20 °C and 6 bar of pressure. The feed solution was an aqueous solution of AO at 20 mg·L^-1^. Three samples per membrane were measured: the column height (in the case of permeances) and the dot position (in the case of rejections) represent the average value, while the error bars represent the standard deviation.

Figure 8 shows the performances of all membranes synthesized (during 6 h of experiment), measured in terms of water permeance and AO rejection. AO with a low molecular weight (265 Da) is a suitable molecule to test the separation ability of these membranes, and rejections around 90% will suggest high molecular weight cut-off. It is important to mention that in the first hour of experiment, all membranes showed relatively low AO rejection and high water permeances, along with high standard deviations in both parameters. These values are related to a transitional regime, where phenomena such as compression and fouling change the surface properties of the membrane. Nevertheless, after the second hour of experiment and onward, both parameters tend to stabilize (steady-state regime) and reach values more commonly found in defect-free nanofiltration membranes. As shown in Figure 8, the water permeance values were higher through the TFC_in membrane than through the TFC_out membrane at any test time (2.2 ± 0.2 and 0.13 ± 0.02 L·m^–2^·h^–1^·bar^–1^ at 6 h, respectively). These membranes were tested in a previous investigation,^25^ obtaining similar permeance values as that predicted in a COMSOL simulation. The research led to the conclusion that the differences between the outer and inner surface morphologies played a critical role in the properties of the PA thin films: the outer PA film permeance is significantly lower than that of the inner. On the contrary, there were a few differences in the rejection values: at 6 h of the filtration experiment, the rejection value obtained by the TFC_in membrane (91 ± 4%) was slightly higher than that obtained by the TFC_out (84 ± 1%) membrane.

When the TFN membrane performances were studied, the differences between the inner and outer composite membranes were maintained at 6 h of operation, favoring the inner configuration membrane: 2.8 ± 0.2 L·m^–2^·h^–1^·bar^–1^ for the TFN_in membrane and 1.0 ± 0.7 L·m^–2^·h^–1^·bar^–1^ for the TFN_out. In addition, the behavior of the TFN_in membrane seems to be more predictable than that of the TFN_out membrane in terms of water permeation because its standard deviation is lower. Rejections at the same conditions, once again, were not significantly different (87 ± 3 and 90 ± 1%, for TFN_in and TFN_out, respectively). These, up to ∼3 h, are non-steady-state results with the permeances reaching a steady state at 4–6 h. Finally, even if steady state was reached in ∼2 h, the results at 2 and 3 h present some fluctuations within the experimental error; as stated in the Figure 8 caption, the error bars were calculated from three different membrane samples, which is proof of the reliability of membrane preparation and nanofiltration testing.

According to previous studies where MIL-101(Cr) was used as filler in TFN membranes, this MOF enhances water permeance due to its specific surface of approximately 2600 m^2^·g^–1^, high porosity (pore apertures of 1.2–1.6 nm and cavities of 2.9–3.4 nm), and hydrophilic character.^6,29^ In these studies, the TFN-MIL-101(Cr) flat sheet membrane was 1.2 times more permeable than the TFC flat sheet membrane.^6^ Here, with a hollow fiber configuration, membrane TFN_out is around 5.5 times more permeable than its corresponding TFC membrane, as shown in Figure 8. Nevertheless, membrane TFN_in is only around 1.4 times more permeable than its corresponding TFC membrane but approximately 21 times more permeable than the TFC_out membrane. These differences suggest that there is a much lower concentration of MOF in the thin layer synthesized on the lumen of the HF than on the outer HF membrane and on the flat membrane fabricated by Sorribas et al.^6^

The XPS tests confirmed that the TFN_out membrane had a much higher concentration of Cr atoms in its PA thin film than the TFN_in membrane (1.8% compared to 0.2%), as Table 3 shows. In addition, as the ED characterization proved that the Cr content is only related to the presence of MIL-101(Cr) NPs, the higher Cr content, the higher the MOF NP content is (see the estimation of MOF content in the nanocomposite membranes synthesized in Table 3). Unexpectedly, lowering the MIL-101(Cr) content had insignificant consequences on the cross-linking degree of the PA layers: as the Cr concentration increases in the reaction, the cross-linking degree of the PA, represented by the C/N and O/N ratios, barely changes (see Table 3). The O/N ratio is especially interesting to estimate the cross-linking degree because it is possible to calculate the proportion of MPD-TMC pairs of the PA that are cross-linked using that ratio.^33^ From this calculation, it can be concluded that a fully cross-linked PA corresponds to an O/N ratio equal to 1, while a fully linear PA has an O/N ratio of 2. Having O/N ratios above 2, as shown in Table 3, would mean that the PA is barely cross-linked. Moreover, the O/N ratio could have been increased due to the presence of MIL-101(Cr) with oxygen in its composition, in agreement with is empirical formula [Cr_3_(O)(OH)(terephthalate)_3_(H_2_O)_2_]·*n*H_2_O.^24^ In contrast to these O/N ratio values, both TFN_in and TFN_out membranes seemed to work properly in the nanofiltration test; in consequence, the PA thin film can be considered correctly formed.

**Table 3:** C/N and O/N Ratios, Cr Atomic Content, and Estimated MOF Content in %mol of the Two Different Nanocomposite Membranes Synthesized

membrane	C/N	O/N	Cr (%atomic)	MOF (%mol)
TFN_in	10.2	2.8	0.2	0.07
TFN_out	10.7	3.0	1.8	0.7

According to the literature, NPs used as fillers influence the cross-linking degree in the polyamide due to their bare presence. NPs, and more importantly, NP agglomerates hinder the TMC and MPD reaction to further lengthen the PA chains because the monomers diffusion paths prior to reaction are less accessible.^34^ This is in agreement with a previous study of Xu et al.^22^ in 2016, where relatively small amounts of MIL-101(Cr) were used to fabricate TFN membranes. They observed that as the amount of MIL-101(Cr) NPs added to the PA thin film increases, the cross-linking degree decreases. In the present article, it is not possible to see any tendency, as the support under each thin film is different, and therefore the MOF content is not the only parameter that changes.

In any event, it is important to highlight that regardless of the MOF content, both thin films are evenly formed and likely cross-linked. This is important to know because, as mentioned in the literature,^22^ lower cross-linking degrees implies more carboxylic acid groups from the TMC present in the PA mass, as they did not react with the MPD molecules, and consequently a more hydrophilic thin film. Therefore, even though it is difficult to measure the contact angle on the lumen of an HF with an ID of 250 μm, both TFN_out and TFN_in membranes are likely hydrophilic with the second membrane type included a much lower quantity of MOF NPs.

Using HFs with an ID so small has numerous advantages, all of them given by the microfluidic regime that takes place inside when a fluid flows through. Such highly ordered flow favors the controlled deposition of NPs on the thin film as it is being formed. This facilitates a good dispersion of MOF NPs in the membrane and, in consequence, a homogeneous influence of them in the PA performance (lower cross-linking degree and a likely higher hydrophilicity).

#### Comparison between Different Synthesis Methods

The synthesis process of the TFN_out membranes is similar to that applied to the flat membranes carried out elsewhere: IP in a static bath. This method led to a higher amount of MOF NPs in the PA films since they end up embedded in the polymer by bare precipitation (even relatively big agglomerates as it was reported in previous researches^29,34,35^). However, several authors have widely evidenced that the substrate used for the IP has a powerful effect on the thin film characteristics. In this way, as Figure 8 shows, the TFC and TFN-MIL-101(Cr) flat membranes had relatively low performances in terms of solvent permeance (0.5 and 0.6 L·m^–2^·h^–1^·bar^–1^, respectively), although the highest in terms of rejection (>99%).^6^ TFN_out membranes were around twice more permeable than TFN flat membranes, and both TFC_in and TFN_in membranes were far more permeable than the two previous configurations. These differences do not fully lay on the MOF NP content since the support used for the flat membranes was an ultrafiltration tailor-made P84 support, the solvent filtrated was methanol, and the solute was a mixture of styrene oligomers with different molecular masses in the range of NF. However, it can give an idea of the meaning of having a TFC HF membrane as the TFN_in that separates water and a solute of 265 Da at 2.8 ± 0.2 L·m^–2^·h^–1^·bar^–1^ with a rejection of 87.5% with no post-treatment or activation method needed.

**Figure 9:**
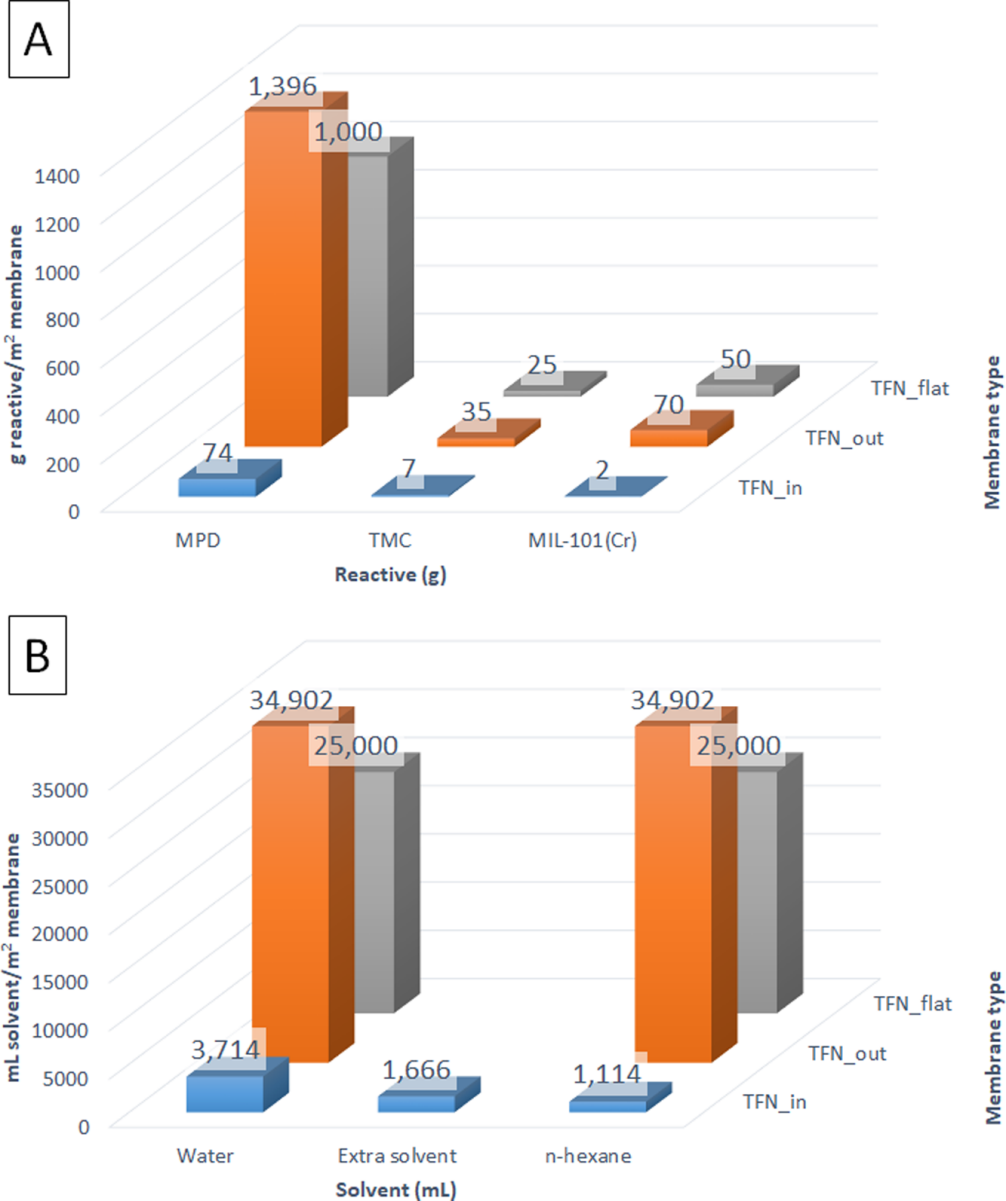
Usage of reactants (MPD, TMC, and MIL-101(Cr)) in the fabrication of the three types of TFN membranes considered in this publication (A). Usage of solvents (water, extra solvent, which in this case is *c*-hexane, and *n*-hexane) in the fabrication of the same three types of TFN membranes considered (B). Numbers in columns represent the value of each column according to the *Y* axis, which would be g reactive/m^2^ membrane in (A), and mL solvent/m^2^ membrane in B.

The application of hollow fibers as supports also allows using less quantity of reactants and solvents to synthesize TFC and TFN membranes (see Figure 9A and Figure 9B). According to the calculations per m^2^ of the membrane surface, the TFN_in membrane would be the cheapest and most cost-effective membrane to be fabricated, thanks to the microfluidic regime.^26,36^ Far more expensive are the TFN flat membrane and the TFN_out membrane, even though the methods to fabricate either of them are potentially optimizable, as it has been the case for the flat configuration.^37^ However, the main advantage of the TFN_in membrane synthesis method is that it is easily scalable because of the highly controllable nature of the low-diameter hollow fibers (overall those in the microfluidic region, with diameters below 500 μm,^36^ as the hollow fiber supports used in this current work) and that a small quantity of MOF in the thin film can significantly enhance its performance.^26,36,38^

**Figure 10:**
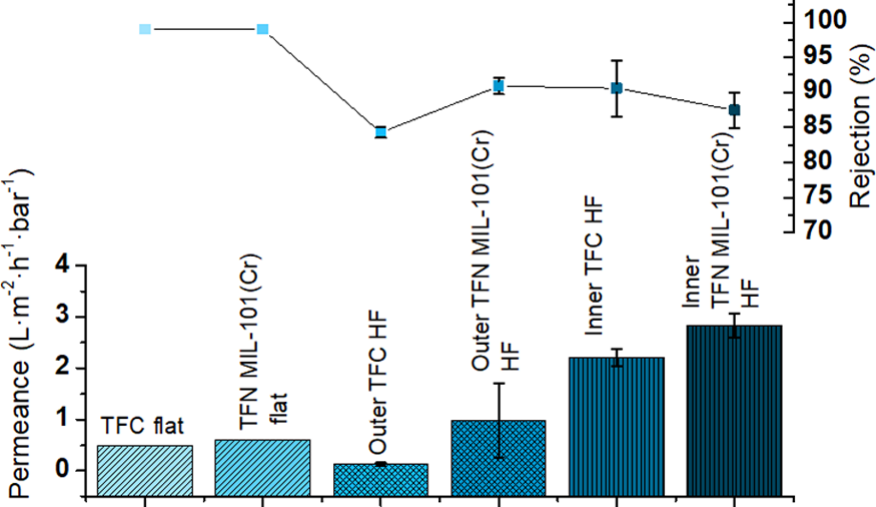
Performance of a TFC flat membrane and TFN MIL-101(Cr) flat membranes (both with methanol as solvent, styrene oligomers as solutes, and P84 as support)^6^ compared to the performances of the TFC_out and TFN_out membranes and TFC_in and TFN_in membranes (water as solvent, AO as solute, and PSf as support).

Knowing the potential of the PSf HF used as a support for this investigation in terms of the m^2^·m^–3^ ratio (6900 m^2^·m^–3^), a TFN MIL-101(Cr) membrane with the thin film synthesized on the inner layer would be more productive than the corresponding TFN flat and TFN_out membranes. The first could offer a lower m^2^·m^–3^ ratio, while the latter has the second lowest permeance of all membranes considered here (see Figure 10).

Finally, the MIL-101(Cr) MOF stability was not addressed in this work focused on establishing a methodology for the preparation of TFN membranes with this MOF. However, previous published results suggest that MIL-101(Cr) is an adequate material for TFN membranes. In fact, several authors have conducted liquid phase stability studies concluding that the MIL-101(Cr) phase preserved its crystallinity in water for 14 days,^39^ and even for 2 months in the 2–12 range of pH.^40^ In addition, mixed matrix membranes containing MIL-101(Cr) showed a stable performance for 4 days under esterification conditions with no evidence of metal leaching.^41^

## Conclusions

The STEM, TEM imaging, EDX, and XPS tests showed that both TFN_in and TFN_out membranes had MIL-101(Cr) NPs embedded in their thin films. However, the IP method used for the TFN_out membrane fabrication allowed to embed more MIL-101(Cr) NPs in its thin film, as the nanofiltration tests evidenced. In consequence, there was a bigger improvement in the water permeance when adding MIL-101(Cr) to the outer thin film than the inner thin film, compared to their corresponding TFC membranes. However, the most permeable membrane was the TFN_in membrane. The reason behind the better performance of the TFN_in membrane may be the roughness and superficial pore sizes of the lumen side of the hollow fiber support.

Interestingly, the TFN_in membrane was even more permeable than its flat membrane version, even though that one shows higher solute rejections. However, rejections were obtained with small molecular weight (265 Da) AO dye, what suggests that the HF membranes would exhibit good performance with other typical solutes with higher molecular weight than AO, while favoring of the intrinsic high water permeance developed here as compared to flat membranes. Besides, the microfluidic regime, present in the fabrication of the composite thin film on the lumen of the hollow fiber, allowed to use much less quantities of reactants and solvents, together with a more gently controlled washing, than the flat membrane or the TFN_out membrane synthesis. The minor use of MOF is particularly attributed to the fact that the laminar flow allows a better reaction control of the interfacial polymerization. In consequence, the TFN_in, with a MOF content approximately ten times lower than that of the TFN_out, achieves a similar degree of crosslinking, which provides a high solute rejection, together with a clear increase in water permeance, which can be attributed to a more even dispersion of MOF nanoparticles. In this way, the MOF is more efficiently applied in TFN_in than in TFN_out membrane, probably producing a less thick polyamide membrane. Finally, even if some TFC or TFN flat membranes in the literature could offer better permeance-rejection binomial than the membranes developed here, the MIL-101(Cr) based TFN membrane has the advantage of being fabricated on a commercial hollow fiber substrate with higher surface to volume (ca. 6900 m^2^·m^-3^) ratio than flat membranes. This would make possible the fabrication of membrane modules with high water flux per m^3^ of equipment.

## Supporting Information Available

(Figure S1) Setup for the continuous nanofiltration, (Figure S2) AFM images of TFN_out and TFN_in hollow fiber membranes, and (Figures S3 and S4) STEM images of TFN_out and TFN_in samples together with their EDS spectra (PDF)

## References

[ref1] Yang Z.; Zhou Y.; Feng Z.; Rui X.; Zhang T.; Zhang Z. A Review on Reverse Osmosis and Nanofiltration Membranes for Water Purification. Polymer 2019, 11, 1252 10.3390/polym11081252. PMC6723865 31362430

[ref2] Hermans S.; Mariën H.; Van Goethem C.; Vankelecom I. F. J. Recent Developments in Thin Film (Nano) Composite Membranes for Solvent Resistant Nanofiltration. Curr. Opin. Chem. Eng. 2015, 8, 45–54. 10.1016/j.coche.2015.01.009.

[ref3] Vandezande P.; Gevers L. E. M.; Vankelecom I. F. J. Solvent Resistant Nanofiltration: Separating on a Molecular Level. Chem. Soc. Rev. 2008, 37, 365–405. 10.1039/b610848m. 18197351

[ref4] Cadotte J. E.; Petersen R. J.; Larson R. E.; Erickson E. E. A new thin-film composite seawater reverse osmosis membrane. Desalination 1980, 32, 25–31. 10.1016/s0011-9164(00)86003-8.

[ref5] Jeong B.-H.; Hoek E. M. V.; Yan Y.; Subramani A.; Huang X.; Hurwitz G.; Ghosh A. K.; Jawor A. Interfacial Polymerization of Thin Film Nanocomposites: A New Concept for Reverse Osmosis Membranes. J. Membr. Sci. 2007, 294, 1–7. 10.1016/j.memsci.2007.02.025.

[ref6] Sorribas S.; Gorgojo P.; Téllez C.; Coronas J.; Livingston A. G. High Flux Thin Film Nanocomposite Membranes Based on Metal-Organic Frameworks for Organic Solvent Nanofiltration. J. Am. Chem. Soc. 2013, 135, 15201–15208. 10.1021/ja407665w. 24044635

[ref7] Ma D.; Peh S. B.; Han G.; Chen S. B. Thin-Film Nanocomposite (TFN) Membranes Incorporated with Super-Hydrophilic Metal- Organic Framework (MOF) UiO-66: Toward Enhancement of Water Flux and Salt Rejection. ACS Appl. Mater. Interfaces 2017, 9, 7523–7534. 10.1021/acsami.6b14223. 28186405

[ref8] Paseta L.; Navarro M.; Coronas J.; Téllez C. Greener Processes in the Preparation of Thin Film Nanocomposite Membranes with Diverse Metal-Organic Frameworks for Organic Solvent Nanofiltration. J. Ind. Eng. Chem. 2019, 77, 344–354. 10.1016/j.jiec.2019.04.057.

[ref9] Echaide-Górriz C.; Navarro M.; Téllez C.; Coronas J. Simultaneous Use of MOFs MIL-101(Cr) and ZIF-11 in Thin Film Nanocomposite Membranes for Organic Solvent Nanofiltration. Dalton Trans. 2017, 46, 6244–6252. 10.1039/c7dt00197e. 28443865

[ref10] Echaide-Górriz C.; Zapata J. A.; Etxeberría-Benavides M.; Téllez C.; Coronas J. Polyamide/MOF Bilayered Thin Film Composite Hollow Fiber Membranes with Tuned MOF Thickness for Water Nanofiltration. Sep. Purif. Technol. 2019, 236, 116265 10.1016/j.seppur.2019.116265.

[ref11] Parthasarathy A.; Brumlik C. J.; Martin C. R.; Collins G. E. Interfacial Polymerization Of Thin Polymer-Films Onto The Surface Of A Microporous Hollow-Fiber Membrane. J. Membr. Sci. 1994, 94, 249–254. 10.1016/0376-7388(93)e0206-y.

[ref12] Liu T.-Y.; Liu Z.-H.; Zhang R.-X.; Wang Y.; Van der Bruggen B.; Wang X.-L. Fabrication of a Thin Film Nanocomposite Hollow Fiber Nanofiltration Membrane for Wastewater Treatment. J. Membr. Sci. 2015, 488, 92–102. 10.1016/j.memsci.2015.04.020.

[ref13] An X.; Ingole P. G.; Choi W. K.; Lee H. K.; Hong S. U.; Jeon J. D. Enhancement of Water Vapor Separation Using ETS-4 Incorporated Thin Film Nanocomposite Membranes Prepared by Interfacial Polymerization. J. Membr. Sci. 2017, 531, 77–85. 10.1016/j.memsci.2017.02.045.

[ref14] Rajaeian B.; Rahimpour A.; Tade M. O.; Liu S. Fabrication and Characterization of Polyamide Thin Film Nanocomposite (TFN) Nanofiltration Membrane Impregnated with TiO_2_ Nanoparticles. Desalination 2013, 313, 176–188. 10.1016/j.desal.2012.12.012.

[ref15] Veríssimo S.; Peinemann K.-V.; Bordado J. Thin-Film Composite Hollow Fiber Membranes: An Optimized Manufacturing Method. J. Membr. Sci. 2005, 264, 48–55. 10.1016/j.memsci.2005.04.020.

[ref16] Gai W.; Zhao D. L.; Chung T.-S. Thin Film Nanocomposite Hollow Fiber Membranes Comprising Na+-Functionalized Carbon Quantum Dots for Brackish Water Desalination. Water Res. 2019, 154, 54–61. 10.1016/j.watres.2019.01.043. 30771707

[ref17] Lin Y.; Chen Y.; Wang R. Thin Film Nanocomposite Hollow Fiber Membranes Incorporated with Surface Functionalized HKUST-1 for Highly-Efficient Reverses Osmosis Desalination Process. J. Membr. Sci. 2019, 589, 117249 10.1016/j.memsci.2019.117249.

[ref18] Urper-Bayram G. M.; Sayinli B.; Bossa N.; Ngaboyamahina E.; Sengur-Tasdemir R.; Ates-Genceli E.; Wiesner M.; Koyuncu I. Thin Film Nanocomposite Nanofiltration Hollow Fiber Membrane Fabrication and Characterization by Electrochemical Impedance Spectroscopy. Polym. Bull. 2020, 77, 3411–3427. 10.1007/s00289-019-02905-w.

[ref19] Plisko T. V.; Liubimova A. S.; Bildyukevich A. V.; Penkova A. V.; Dmitrenko M. E.; Mikhailovskii V. Y.; Melnikova G. B.; Semenov K. N.; Doroshkevich N. V.; Kuzminova A. I. Fabrication and Characterization of Polyamide-Fullerenol Thin Film Nanocomposite Hollow Fiber Membranes with Enhanced Antifouling Performance. J. Membr. Sci. 2018, 551, 20–36. 10.1016/j.memsci.2018.01.015.

[ref20] Ingole P. G.; Pawar R. R.; Baig M. I.; Jeon J. D.; Lee H. K. Thin Film Nanocomposite (TFN) Hollow Fiber Membranes Incorporated with Functionalized Acid-Activated Bentonite (ABn-NH) Clay: Towards Enhancement of Water Vapor Permeance and Selectivity. J. Mater. Chem. A 2017, 5, 20947–20958. 10.1039/c7ta04945e.

[ref21] Baig M. I.; Ingole P. G.; Jeon J.; Hong S. U.; Choi W. K.; Lee H. K. Water Vapor Transport Properties of Interfacially Polymerized Thin Film Nanocomposite Membranes Modified with Graphene Oxide and GO-TiO2 Nanofillers. Chem. Eng. J. 2019, 373, 1190–1202. 10.1016/j.cej.2019.05.122.

[ref22] Xu Y.; Gao X.; Wang X.; Wang Q.; Ji Z.; Wang X.; Wu T.; Gao C. Highly and Stably Water Permeable Thin Film Nanocomposite Membranes Doped with MIL-101 (Cr) Nanoparticles for Reverse Osmosis Application. Materials 2016, 9, 870 10.3390/ma9110870. PMC5457272 28773990

[ref23] Ferey G.; Mellot-Draznieks C.; Serre C.; Millange F.; Dutour J.; Surble S.; Margiolaki I. A Chromium Terephthalate-Based Solid with Unusually Large Pore Volumes and Surface Area. Science 2005, 309, 2040–2042. 10.1126/science.1116275. 16179475

[ref24] Khan N. A.; Kang I. J.; Seok H. Y.; Jhung S. H. Facile Synthesis of Nano-Sized Metal-Organic Frameworks, Chromium-Benzenedicarboxylate, MIL-101. Chem. Eng. J. 2011, 166, 1152–1157. 10.1016/j.cej.2010.11.098.

[ref25] Echaide-Górriz C.; Malankowska M.; Téllez C.; Coronas J. Nanofiltration Thin Film Composite Membrane on Either the Internal or the External Surface of a Polysulfone Hollow Fiber. AIChE J. 2020, 66, e16970 10.1002/aic.16970.

[ref26] Cacho-Bailo F.; Catalán-Aguirre S.; Etxeberría-Benavides M.; Karvan O.; Sebastian V.; Téllez C.; Coronas J. Metal-Organic Framework Membranes on the Inner-Side of a Polymeric Hollow Fiber by Microfluidic Synthesis. J. Membr. Sci. 2015, 476, 277–285. 10.1016/j.memsci.2014.11.016.

[ref27] Freger V. Swelling and Morphology of the Skin Layer of Polyamide Composite Membranes: An Atomic Force Microscopy Study. Environ. Sci. Technol. 2004, 38, 3168–3175. 10.1021/es034815u. 15224751

[ref28] Freger V. Nanoscale Heterogeneity of Polyamide Membranes Formed by Interfacial Polymerization. Langmuir 2003, 19, 4791–4797. 10.1021/la020920q.

[ref29] Echaide-Górriz C.; Sorribas S.; Téllez C.; Coronas J. MOF Nanoparticles of MIL-68(Al), MIL-101(Cr) and ZIF-11 for Thin Film Nanocomposite Organic Solvent Nanofiltration Membranes. RSC Adv. 2016, 6, 90417–90426. 10.1039/c6ra17522h.

[ref30] Cravillon J.; Múnzer S.; Lohmeier S.-J.; Feldhoff A.; Huber K.; Wiebcke M. Rapid Room-Temperature Synthesis and Characterization of Nanocrystals of a Prototypical Zeolitic Imidazolate Framework. Chem. Mater. 2009, 21, 1410–1412. 10.1021/cm900166h.

[ref31] Park K. S.; Ni Z.; Cote A. P.; Choi J. Y.; Huang R.; Uribe-Romo F. J.; Chae H. K.; O’Keeffe M.; Yaghi O. M. Exceptional Chemical and Thermal Stability of Zeolitic Imidazolate Frameworks. Proc. Natl. Acad. Sci. U. S. A. 2006, 103, 10186–10191. 10.1073/pnas.0602439103. 16798880 PMC1502432

[ref32] Ghosh A. K.; Hoek E. M. V. Impacts of Support Membrane Structure and Chemistry on Polyamide-Polysulfone Interfacial Composite Membranes. J. Membr. Sci. 2009, 336, 140–148. 10.1016/j.memsci.2009.03.024.

[ref33] Zhao Y.-Y.; Liu Y.-L.; Wang X.-M.; Huang X.; Xie Y. F. Impacts of Metal–Organic Frameworks on Structure and Performance of Polyamide Thin-Film Nanocomposite Membranes. ACS Appl. Mater. Interfaces 2019, 11, 13724–13734. 10.1021/acsami.9b01923. 30874427

[ref34] Duan J.; Pan Y.; Pacheco F.; Litwiller E.; Lai Z.; Pinnau I. High-Performance Polyamide Thin-Film-Nanocomposite Reverse Osmosis Membranes Containing Hydrophobic Zeolitic Imidazolate Framework-8. J. Membr. Sci. 2015, 476, 303–310. 10.1016/j.memsci.2014.11.038.

[ref35] Butler E. L.; Petit C.; Livingston A. G. Poly(Piperazine Trimesamide) Thin Film Nanocomposite Membrane Formation Based on MIL-101: Filler Aggregation and Interfacial Polymerization Dynamics. J. Membr. Sci. 2019, 596, 117482 10.1016/j.memsci.2019.117482.

[ref36] Elvira K. S.; i Solvas X. C.; Wootton R. C. R.; deMello A. J. The Past, Present and Potential for Microfluidic Reactor Technology in Chemical Synthesis. Nat. Chem. 2013, 5, 905–915. 10.1038/nchem.1753. 24153367

[ref37] Navarro M.; Benito J.; Paseta L.; Gascón I.; Coronas J.; Téllez C. Thin-Film Nanocomposite Membrane with the Minimum Amount of MOF by the Langmuir-Schaefer Technique for Nanofiltration. ACS Appl. Mater. Interfaces 2018, 10, 1278–1287. 10.1021/acsami.7b17477. 29243908

[ref38] Echaide-Górriz C.; Clément C.; Cacho-Bailo F.; Téllez C.; Coronas J. New Strategies Based on Microfluidics for the Synthesis of Metal-Organic Frameworks and Their Membranes. J. Mater. Chem. A 2018, 6, 5485–5506. 10.1039/c8ta01232f.

[ref39] Du P. D.; Thanh H. T. M.; To T. C.; Thang H. S.; Tinh M. X.; Tuyen T. N.; Hoa T. T.; Khieu D. Q. Metal-Organic Framework MIL-101: Synthesis and Photocatalytic Degradation of Remazol Black B Dye. J. Nanomater. 2019, 2019, 1–15. 10.1155/2019/6061275.

[ref40] Leus K.; Bogaerts T.; De Decker J.; Depauw H.; Hendrickx K.; Vrielinck H.; Van Speybroeck V.; Van Der Voort P. Systematic Study of the Chemical and Hydrothermal Stability of Selected “Stable” Metal Organic Frameworks. Microporous Mesoporous Mater. 2016, 226, 110–116. 10.1016/j.micromeso.2015.11.055.

[ref41] de la Iglesia O.; Sorribas S.; Almendro E.; Zornoza B.; Tellez C.; Coronas J. Metal-organic framework MIL-101(Cr) based mixed matrix membranes for esterification of ethanol and acetic acid in a membrane reactor. Renewable Energy 2016, 88, 12–19. 10.1016/j.renene.2015.11.025.

