# Supplementary material for: Polyamide-MIL-101(Cr) Thin Films Synthesized on Either the Outer or Inner Surfaces of a Polysulfone Hollow Fiber for Water Nanofiltration
Source: ACS Appl Mater Interfaces. 2021 Feb 3;13(6):7773–83. doi: 10.1021/acsami.0c21571 (PMC8892444; doi:10.1021/acsami.0c21571)
Supplement: Supplementary file 1 — am0c21571_si_001.pdf [file am0c21571_si_001.pdf]

SUPPORTING INFORMATION

Polyamide-MIL-101(Cr) thin films synthesized on either the outer or inner surfaces of a polysulfone hollow fiber for water nanofiltration

Carlos Echaide-Górriz[a,b,*], Yolanda Aysa-Martínez[a,b], Marta Navarro[c], Carlos Téllez[a,b], Joaquín Coronas[a,b,*]

[a]Instituto de Nanociencia y Materiales de Aragón (INMA), Universidad de Zaragoza-CSIC, 50018 Zaragoza. Spain

[b]Chemical and Environmental Engineering Department, Universidad de Zaragoza, 50018 Zaragoza. Spain

[c] Advanced Microscopy Laboratory (LMA), Universidad de Zaragoza, 50018 Zaragoza, Spain.

*Corresponding authors: C. Echaide-Górriz, cechaide@unizar.es; J. Coronas, coronas@unizar.es.

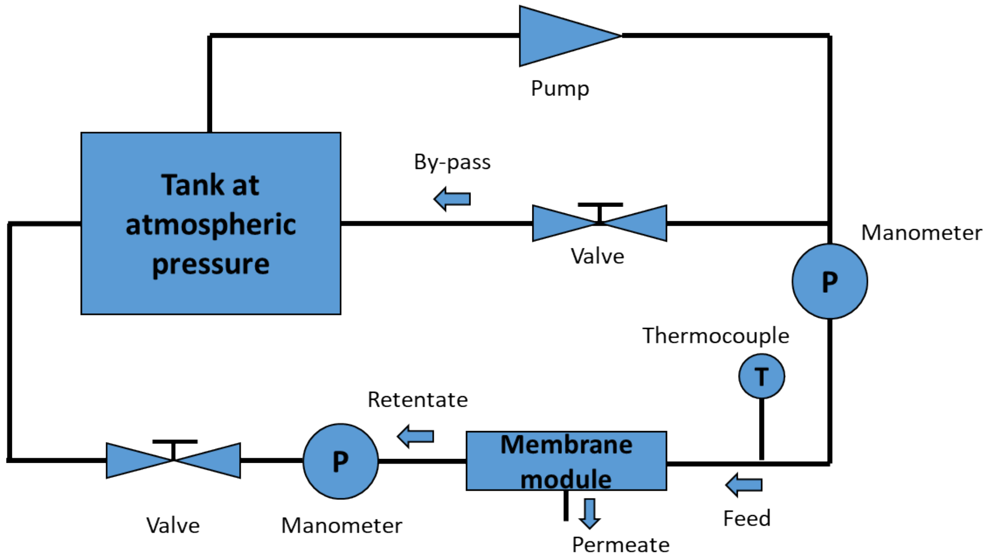

Fig. S1. Installation for the continuous nanofiltration tests.

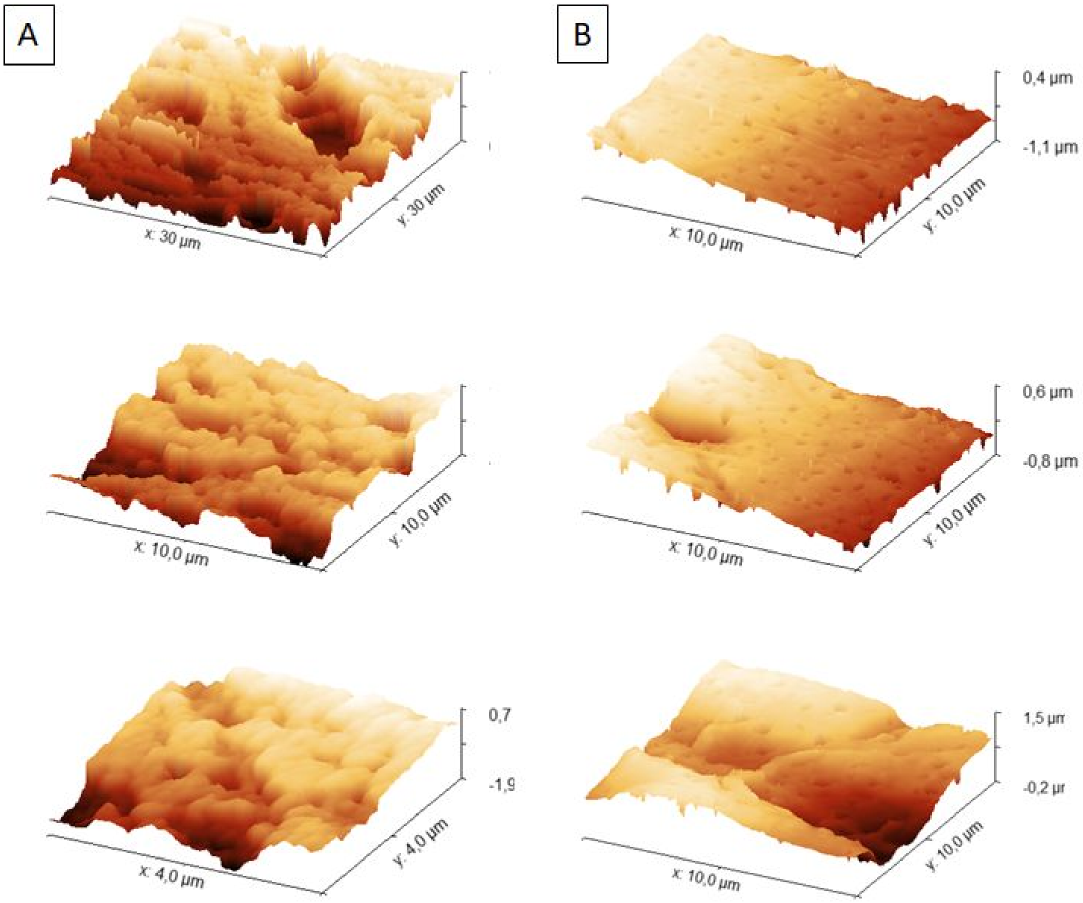

Fig. S2. As obtained by AFM, 3D diagrams of three areas (10x10 µm) of the outer surface of the hollow fiber support (A) and other three areas (30x30 µm, 10x10 µm, and 4x4 µm) of the inner surface of the hollow fiber support (B)

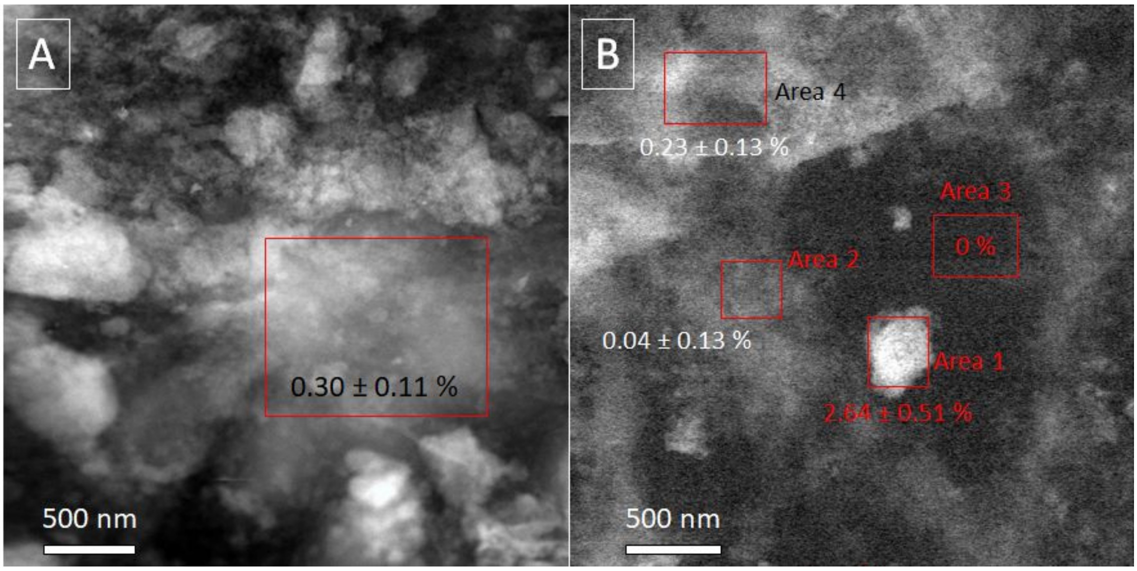

Fig. S3. STEM image of the detached polyamide thin film from the TFN_out membrane with the concentration of Cr atoms in the area analysed (highlighted in red) (A). EDS analysis in the area highlighted in A (B). STEM image of the detached polyamide thin film from the TFN_in membrane with the concentration of Cr atoms in the areas analysed (highlighted in red) (B).

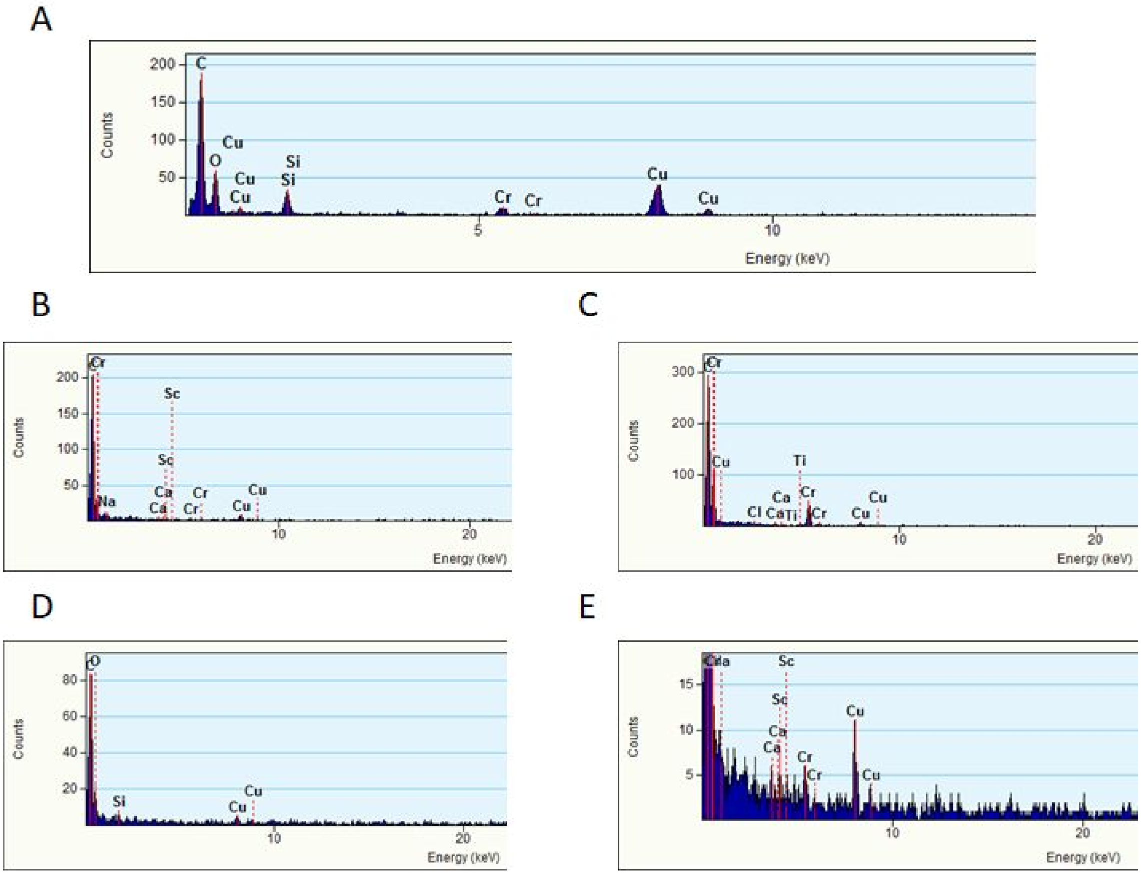

*Fig. S4. EDS spectra of the area analysed on Fig. S3A (A), and the areas 1 (B), 2 (C), 3 (D) and 4 (E) on Fig. S3B. *The Si peaks comes from the EDS detector.*
